# Populene D Analogues: Design, Concise Synthesis and Antiproliferative Activity

**DOI:** 10.3390/molecules17089621

**Published:** 2012-08-10

**Authors:** Kachi R. Kishore Kumar Reddy, Giovanna B. Longato, João E. de Carvalho, Ana L. T. G. Ruiz, Luiz F. Silva

**Affiliations:** 1 Instituto de Química, Universidade de São Paulo, Av. Prof. Lineu Prestes, 748, CP 26077, CEP São Paulo 05513-970, SP, Brazil; Email: kishorereddyk@gmail.com; 2 Divisão de Farmacologia e Toxicologia, Centro Pluridisciplinar de Pesquisas Químicas, Biológicas e Agrícolas (CPQBA), UNICAMP, CP6171, Campinas 13083-970, SP, Brazil; Email: giovannabl@yahoo.com.br (G.B.L.); carvalho@cpqba.unicamp.br (J.E.C.)

**Keywords:** osochromene, pyrans, Prins cyclization, iodine, antiproliferative, cancer

## Abstract

An efficient and concise synthesis of nine populene D analogues was performed using an iodine-catalyzed Prins cyclization as the key transformation. The antiproliferative activity of these new pyrans against several cancer cell lines was then investigated. Among them, an isochromene with moderate activity (mean logGI_50_ = 0.91) was found. Additionally, compounds with selectivity toward the tumor cell lines NCI-ADR/RES, OVCAR-3, and HT29 were discovered.

## 1. Introduction

**Figure 1:**
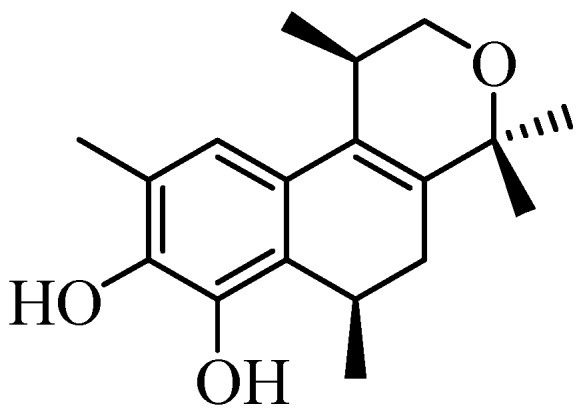
Structure of populene D.

Natural products have always played an important role in drug discovery [1,2,3,4,5]. Although it is not always possible to understand the exact function of the secondary metabolites isolated from natural sources, they are often used as inspiration for new drugs, particularly in the area of cancer [6,7,8,9]. The pyran subunit can be frequently recognized in the structure of numerous natural and synthetic compounds with remarkable biological and pharmacological properties [10,11,12,13]. Populene D (Figure 1) is a natural sesquiterpenoid recently isolated from *Thespesia populnea*, which possesses strong inhibitory activity against several human cancer cell lines: cervical cancer (HeLa, IC_50_ = 1.85 µg/mL) and breast cancer (MCF-7, IC_50_ = 0.95 µg/mL) [14]. Populene D is also active against colon cancer (HT-29, IC_50_ = 2.37 µg/mL), oral cancer (KB, IC_50_ = 3.10 µg/mL) and has antibacterial properties (*B. subtilis*, MIC = 4.69 µg/mL) [14].

**Scheme 1:**
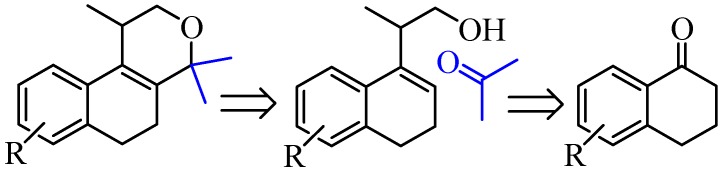
Retrosynthesis for populene D analogues.

We envisioned that populene D analogues could be synthesized by the iodine-catalyzed Prins cyclization of homoallylic alcohols and acetone (Scheme 1) [15]. Considering that several homoallylic alcohols can be easily prepared from commercially available tetralones [16], this route could provide access to analogues of a natural compound with potential anticancer activity. Herein, we describe our first results regarding the concise synthesis of nine isochromenes and their antiproliferative activity against several tumor cell lines.

## 2. Results and Discussion

### 2.1. Synthesis of Populene D Analogues

The homoallylic alcohols **1a–d** were prepared in three steps from commercially available 1-tetralones as previously reported [16]. The Prins cyclization of the homoallylic alcohols **1a–d** and acetone in the presence of iodine gave the methoxy substituted populene D analogues **2a**–**d** in 73–88% yield (Table 1, entries 1–4). The treatment of 2a–c with sodium ethanethiolate [17,18] gave hydroxy substituted populene D analogues 3a–c in 71–82% yield (Table 2, entries 1–3). Under analogous conditions, the dimethoxy substrate **2d** gave the mono deprotected compound **3d** in 42% yield (entry 4). The structure of **3d** was assigned by NMR analysis, including HMBC experiments. The double deprotection to obtain **3e** was achieved using excess of sodium ethanethiolate and longer reaction time (entry 5). In summary, nine new populene D analogues were efficiently prepared using as key reaction a Prins cyclization and fully characterized.

**Table 1:** Iodine-catalyzed Prins cyclization.

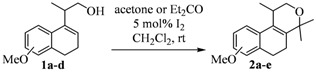		
Entry	Homoallylic alcohols 1a–d	Products 2a–e (yield)
1	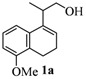	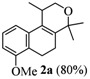
2	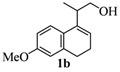	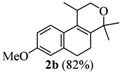
3	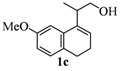	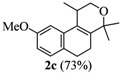
4	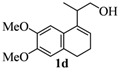	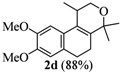

**Table 2:** Cleavage of the ether moiety.

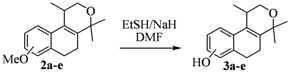		
**Entry**	**Substrates 2a–d**	**Products 3a–e (yield)**
1	**2a**	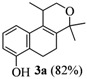
2	**2b**	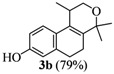
3	**2c**	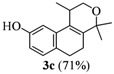
4	**2d**	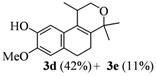
5	**2d**	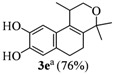

^a^ using excess of sodium ethanethiolate and longer reaction time.

### 2.2. Antiproliferative Activity of Populene D Analogues

The *in vitro* antiproliferative activity of populene analogues **2a**–**e** and **3a**–**e** was investigated toward nine human tumor cell lines [CNS (U251), breast (MCF-7), ovarian (NCI-ADR/RES, OVCAR-03), renal (786-0), non-small cell lung (NCI-H460), prostate (PC-3), colon (HT-29) and leukemia (k-562)] and one human normal cell line (HaCat, human keratinocytes). The populene D analogues were tested at concentrations between 0.25 and 250 µg/mL and doxorubicin (DOX 0.025–25 µg/mL) was used as positive control. Two effective concentrations, eliciting 50% growth inhibition (GI_50_) and total growth inhibition (TGI), were determined after 48 h-cell treatment (Table 3 and Table 4). To analyze the GI_50 _parameter, mean log GI_50_ was calculated by conversion of the GI_50_ values for each tumor cell line tested (not including the normal cell line HaCat) against a test compound and then these values were averaged. According to National Cancer Institute (NCI/EUA), if mean logGI_50_ < 1.50 a tested compound could be considered as active and might be classified as of weak (1.1 < mean logGI_50_ < 1.5), moderate (0 < mean logGI_50_ < 1.1) or potent (mean logGI_50_ < 0) activity [19]. Using these criteria, phenol **3e** (mean logGI_50_ = 0.91) was the most active populene analogue synthetized, presenting a moderate cytostatic activity with low selective against human cell lines evaluated. On the other hand, heterocycles **3c ** and **3d **presented similar general weak cytostatic effect (mean log GI_50_ = 1.15 and 1.16, respectively), but compound **3c** showed a high selectivity to the ovarian cell line OVCAR-3 (GI_50_ = 1.8 µg/mL), whereas pyran **3d** was selective for the ovarian expressing multidrug resistance phenotype (NCI/ADR-RES, GI_50_ = 5.2 µg/mL) and to colon (HT29, GI_50_ = 6.0 µg/mL) cell lines. Isochromenes **3a** and **3b **were considered inactive (mean logGI_50_ > 2.32 and > 2.00, respectively). The methoxy derivatives **2a**–**c **presented a weak cytostatic activity (mean logGI_50_ from 1.12 to 1.23), whereas compound **2d** was inactive. However, the following differences in selectivity were found for these ethers: (i) pyran **2a** is more selective to leukemia (K562, GI_50_ = 3.1 µg/mL); (ii) compound **2b** was more active against colon (HT29, GI_50_ = 6.6 µg/mL) cell line; (iii) heterocycle **2e** presented a slight selectivity to glioma (U251, GI_50_ = 6.3 µg/mL) cell line.

In summary, these results suggest that the hydroxyl/methoxyl pattern of substitution has an important influence in the antiproliferative activity. Moreover, the dihydroxy-substituted populene D analogue **3e** is the most active compound in the **2a**–**d** and **3a**–**e** series. Considering the monosubstituted compounds, the conversion of the methoxy (**2a** and **2b**) into a hydroxy (**3a** and **3b**) group resulted in lower activity, whereas the substitution from **2c** to **3c** led to a slightly increase in the cytostatic activity. Among the disubstituted compounds, the conversion of the methoxy (**2d**) to hydroxy (**3d** and **3e**) increased the activity.

The results obtained for the TGI parameter confirm the GI_50_ evaluation, indicating moderate activity for populene analogues **2a**–**2c** and **3c**–**3e** (Table 4). The evaluation for the normal human cell lines (HaCat) shows GI_50_ and TGI values on the same order of magnitude than for the tumor cell lines, suggesting that populene D analogues may present *in vivo* toxicity, similar to the known chemotherapic drugs.

**Table 3:** Antiproliferative activity (GI_50_, μg/mL) of **2a–d** and **3a–e** on human cell lines.

Cell lines ^a^	Doxorubicine ^b^	2a	2b	2c	2d	3a	3b	3c	3d	3e
**U251**	0.062	25.0	24.6	25.0	>250	>250	46.3	27.8	23.1	19.6
**MCF-7**	0.21	25.0	23.2	25.0	38.8	>250	107.8	24.7	24.6	5.5
**NCI-ADR/RES**	1.3	25.0	26.8	25.0	100.7	>250	>250	25.0	5.2	5.7
**786-0**	0.04	25.0	25.5	25.0	45.8	37.3	36.8	24.6	24.1	5.6
**NCI-H460**	<0.025	N.T.	N.T.	N.T.	N.T.	>250	41.4	24.2	45.1	17.4
**PC-3**	0.27	25.0	27.0	25.0	71.4	>250	>250	24.5	N.T.	N.T.
**OVCAR-3**	0.26	25.0	24.5	25.0	193.4	>250	>250	1.8	24.3	3.9
**HT-29**	0.22	25.0	6.6	25.0	23.1	>250	78.4	22.9	6.0	19.5
**K-562**	0.4	3.1	9.6	25.0	84.6	>250	41.2	8.7	N.T.	N.T.
**HaCat**	0.23	25.0	26.1	25.0	>250	>250	>250	3.6	4.2	4.2
***Mean logGI_50_^c, d^***	*−0.78 P*	*1.17 W*	*1.18 W*	*1.21 W*	*>1.70 I*	*>2.31 I*	*>1.95 I*	*1.22 W*	*1.24 W*	*0.95 M*

N.T.: not tested. ^a^ Tumor cell lines: U251—glioma; MCF-7—mammary; NCI-ADR/RES—drug resistant ovary; 786-0—kidney; NCI-H460—lung; NCI-PC-3—prostate; OVCAR-3—ovary; HT-29 colon; K562—leukemia. Normal cell lines: HaCat—immortalized keratinocytes; ^b^ Positive control; ^c^ Mean logGI_50_: The GI_50_ value for each tumor cell line tested (not including the normal cell line HaCat) against a test compound is converted to its logGI_50_ value and these logGI_50_ values are averaged; ^d^ NCI’s criteria [19]: I: mean logGI_50_ > 1.5 = inactive; W, weak activity: 1.1 < mean logGI_50_ < 1.5; M, moderate activity: 0 < mean logGI_50_ < 1.1; P, potent activity: mean log GI_50_ < 0.

**Table 4:** Total growth inhibition (TGI, μg/mL) of 2a–d and 3a–e on human cell lines.

Cell lines ^a^	Doxorubicine ^b^	2a	2b	2c	2d	3a	3b	3c	3d	3e
**U251**	1.1	52.0	53.5	40.0	>250	>250	>250	67.9	45.9	28.4
**MCF-7**	8.8	51.3	46.9	45.3	>250	>250	>250	45.0	58.2	41.6
**NCI-ADR/RES**	25.0	83.5	104.4	66.3	>250	>250	>250	199.5	37.6	>250
**786-0**	0.62	50.6	44.0	36.1	>250	>250	>250	43.0	47.8	19.4
**NCI-H460**	0.025	N.T.	N.T.	N.T.	N.T.	>250	>250	45.9	>250	>250
**PC-3**	4.4	64.6	41.4	69.2	>250	>250	>250	51.8	N.T.	N.T.
**OVCAR-3**	3.9	52.3	56.2	67.0	>250	>250	>250	31.5	69.9	34.4
**HT-29**	25.0	39.1	33.0	41.8	>250	>250	>250	53.3	32.3	39.0
**K-562**	25.0	>250	>250	128.1	>250	>250	>250	39.2	N.T.	N.T.
**HaCat**	0.67	66.9	54.4	32.0	>250	>250	>250	>250	160.7	>250

N.T.: not tested; ^a^ Tumor cell lines: U251—glioma; MCF-7—mammary; NCI-ADR/RES—drug resistant ovary; 786-0—kidney; NCI-H460—lung; NCI-PC-3—prostate; OVCAR-3—ovary; HT-29 colon; K562—leukemia. Normal cell lines: HaCat—immortalized keratinocytes. ^b^ Positive control.

## 3. Experimental

### 3.1. General

All commercially available reagents were used without further purification unless otherwise noted. CH_2_Cl_2_ and DMF were freshly distilled over CaH_2_. TLC analyses were performed in silica gel plates, using UV and/or *p*-anisaldehyde solution for visualization. Flash column chromatography was performed using silica gel 200–400 mesh. Melting points are uncorrected. All NMR analyses were recorded using CDCl_3_ as solvent and TMS as internal pattern in Bruker (AC200) or Varian (INOVA300) spectrometers. IR spectra were measured on a Perkin-Elmer 1750-FT. HRMS analysis were performed on a Bruker Daltonics Microtof Eletrospray. Melting points were recorded on a Buchi R-535 apparatus and are uncorrected. The homoallylic alcohols **1a–c** were prepared in three steps from commercially available 1-tetralones as previously reported [16].

### 3.2. General Procedure for Prins Cyclizations

To a stirred solution of **1a–d** (1.0 mmol) and (CH_3_)_2_CO (1.2 mmol) in CH_2_Cl_2_ (5 mL), was added I_2_ (0.050 mmol). After 2 h, Na_2_SO_3_ (0.60 mmol) and H_2_O (10 mL) were added. The aqueous phase was extracted with AcOEt (3 × 5 mL). The combined organic phase was washed with brine (5 mL) and dried over anhydrous MgSO_4_. The solvent was removed under reduced pressure. The crude product was purified by flash column chromatography (5% AcOEt in hexanes), affording **2a–d**.

*4,5,6-Tetrahydro-7-methoxy-1,4,4-trimethyl-1H-benzo[f]isochromene* (**2a**). Yield: 80%. White solid; mp: 65–67 °C; IR (film): 3073, 2969, 1574, 1470, 1460, 784 cm^−1^; ^1^H-NMR (200 MHz, CDCl_3_) δ: 1.23 (d, *J *= 6.8 Hz, 3H), 1.32 (s, 3H), 1.42 (s, 3H), 2.05–2.25 (m, 2H), 2.48–2.66 (m, 2H), 2.84–2.99 (m, 1H), 3.65 (dd, *J *= 2.4, 11.1 Hz, 1H), 3.84 (s, 3H), 3.93 (dd, *J *= 3.3, 11.1 Hz, 1H), 6.78 (d, *J *= 8.3 Hz, 1H), 6.94 (d, *J *= 7.8 Hz, 1H), 7.19 (dd, *J *= 7.8, 8.2 Hz, 1H); ^13^C-NMR (50 MHz, CDCl_3_) δ: 18.2, 20.4, 23.9, 24.1, 27.9, 29.2, 55.5, 65.8, 74.8, 109.0, 115.3, 123.6, 126.4, 129.6, 135.2, 138.6, 155.9; LRMS *m/z *(rel. int.): 258 (M^+^, 9.1), 244 (13), 243 (100), 227 (15), 105 (35), 77 (53), 43 (66); HRMS [ESI(+)] calcd. for [C_17_H_22_O_2_+ H]^+^ 259.1693, found 259.1679.

*2,4,5,6-Tetrahydro-8-methoxy-1,4,4-trimethyl-1H-benzo[f]isochromene* (**2b**). Yield: 82%. White solid; mp: 89.5–91.3 °C; IR (film): 3005, 2970, 1610, 1570, 1492, 1427, 670, 612 cm^−1^; ^1^H-NMR (200 MHz, CDCl_3_) δ: 1.21 (d, *J *= 6.8 Hz, 3H), 1.29 (s, 3H), 1.39 (s, 3H), 203–2.15 (m, 2H), 2.56–2.72 (m, 3H), 3.62 (dd, *J *= 2.2, 11.1 Hz, 1H), 3.78 (s, 3H), 3.90 (dd, *J *= 3.3, 11.1 Hz, 1H), 6.69–6.75 (m, 2H), 7.16 (d, *J *= 8.3 Hz, 1H); ^13^C-NMR (50 MHz, CDCl_3_) δ: 18.0, 24.0, 24.3, 27.9, 28.9, 29.0, 55.0, 65.6, 74.6, 110.9, 113.4, 123.3, 126.9, 129.3, 135.7, 137.4, 157.9; LRMS *m/z *(rel. int.): 258 (M^+^, 9.9), 244 (13), 243 (100); Elemental analysis calcd. for [C_17_H_22_O_2_] C 79.03, H 8.58, found C 78.61, H 8.37.

*2,4,5,6-Tetrahydro-9-methoxy-1,4,4-trimethyl-1H-benzo[f]isochromene* (**2c**). Yield: 73%. Colorless viscous oil; IR (film): 3076, 2969, 1605, 1573, 1496, 1461, 835, 803 cm^−1^; ^1^H-NMR (200 MHz, CDCl_3_) δ: 1.23 (d, *J *= 6.8 Hz, 3H), 1.30 (s, 3H), 1.40 (s, 3H), 2.03–2.16 (m, 2H), 2.59–2.69 (m, 3H), 3.63 (dd, *J *= 2.4, 11.2 Hz, 1H), 3.79 (s, 3H), 3.91 (dd, *J *= 3.2, 11.2 Hz, 1H), 6.67 (dd, *J *= 2.4, 8.1 Hz, 1H), 6.84 (d, *J *= 2.4 Hz, 1H), 7.03 (d, *J *= 8.1 Hz, 1H); ^13^C-NMR (50 MHz, CDCl_3_) δ: 18.0, 24.1, 24.9, 27.7, 27.8, 28.9, 55.2, 65.7, 74.8, 109.3, 110.3, 127.8, 128.0, 129.7, 135.1, 139.2, 158.4; LRMS *m/z *(rel. int.): 258 (M^+^, 11.5), 244 (14), 243 (100); HRMS [ESI(+)] calcd. for [C_17_H_22_O_2_+ H]^+^ 259.1693, found 259.1699.

*2,4,5,6-Tetrahydro-8,9-dimethoxy-1,4,4-trimethyl-1H-benzo[f]isochromene * (**2d**). Yield: 81%. White solid; mp: 115–117 °C; IR (film): 2968, 2931, 1512, 1238, 1203, 856, 806 cm^−1^; ^1^H-NMR (200 MHz, CDCl_3_) δ: 1.24 (d, *J *= 6.8 Hz, 3H), 1.30 (s, 3H), 1.40 (s, 3H), 2.04–2.17 (m, 2H), 2.58–2.70 (m, 3H), 3.64 (dd, *J *= 2.2, 11.2 Hz, 1H), 3.88 (s, 3H), 3.90–3.96 (m, 1H), 6.69 (s, 1H), 6.83 (s, 1H); ^13^C-NMR (50 MHz, CDCl_3_) δ: 18.1, 24.1, 24.7, 28.0,28.3, 29.1, 55.9, 56.2, 65.6, 74.8, 106.9, 111.2, 126.7, 128.5, 129.2, 136.4, 147.2, 147.3; LRMS *m/z *(rel. int.): 288 (M^+^, 22), 274 (17), 273 (100); CH analysis calcd. for [C_18_H_24_O_3_] C 74.97, H 8.39, found C 74.77, H 8.75.

### 3.3. General Procedure for the Deprotections of 3a–d

Under an inert atmosphere of N_2_, NaH (17.5 mmol, 60% in mineral oil) was washed with anhydrous hexanes (2 × 10 mL). After a few minutes, anhydrous DMF (5 mL) was added. To this mixture was slowly added a solution of EtSH (12.6 mmol) in anhydrous DMF (0.4 mL) at 0 °C and the resulting yellow gray solution was stirred for 20 min at rt. A solution of **2a–d** (0.39 mmol) in DMF (1 mL) was then added dropwise and the resulting mixture was stirred for 5 h at 140 °C, becoming slightly brown. The mixture was cooled to the rt and a saturated solution of NH_4_Cl (5 mL) was added. The mixture was extracted with Et_2_O (3 × 10 mL) and the organic phase was washed with H_2_O (5 mL), with brine (5 mL), and dried over anhydrous MgSO_4_. The solvent was removed under reduced pressure and the resulting brown oil was purified by flash chromatography (30% AcOEt in hexanes), affording **3a–d**.

*1,4,4-Trimethyl-1,4,5,6-tetrahydro-2H-benzo[f]isochromen-7-ol* (**3a**). Yield: 82%. White solid; mp: 139.2–140.3 °C; IR (film): 3289, 2971, 2932, 1578, 1468, 916, 833 cm^−1^; ^1^H-NMR (200 MHz, CDCl_3_) δ: 1.22 (d, *J *= 6.8 Hz, 3H), 1.32 (s, 3H), 1.42 (s, 3H), 2.05–2.25 (m, 2H), 2.49–2.65 (m, 2H), 2.80–2.91 (m, 1H), 3.64 (dd, *J *= 2.3, 11.1 Hz, 1H), 3.92 (dd, *J *= 3.3, 11.1 Hz, 1H), 6.67 (d, *J *= 0.6, 7.9 Hz, 1H), 6.90 (d, *J* = 7.6 Hz, 1H), 7.09 (dd, *J *= 7.8, 8.0 Hz, 1H); ^13^C-NMR (50 MHz, CDCl_3_) δ: 18.2, 20.5, 23.8, 24.1, 27.9, 29.2, 65.8, 74.8, 113.8, 115.6, 121.2, 126.6, 129.9, 135.6, 138.5, 151.9; LRMS *m/z *(rel. int.): 244 (M^+^, 9.0), 230 (13), 229 (100); CH analysis calcd. for [C_16_H_21_O_2_] C 78.65, H 8.25, found C 78.45, H 8.00.

*1,4,4-Trimethyl-1,4,5,6-tetrahydro-2H-benzo[f]isochromen-8-ol* (**3b**). Yield: 79%. White solid; mp: 199–201 °C; IR (film): 3350, 2968, 2932, 1605, 1502, 1440, 849, 834 cm^−1^; ^1^H-NMR (200 MHz, CDCl_3_) δ: 1.22 (d, *J *= 6.8 Hz, 3H), 1.31 (s, 3H), 1.40 (s, 3H), 2.08–2.16 (m, 2H), 2.57–2.71 (m, 3H), 3.64 (dd, *J *= 2.3, 11.2 Hz, 1H), 3.93 (dd, *J *= 3.2, 11.2 Hz, 1H),5.13 (s, 1H), 6.64–6.70 (m, 2H), 7.12 (d, *J *= 8.1 Hz, 1H); ^13^C-NMR (50 MHz, CDCl_3_) δ: 18.1, 24.1, 24.4, 28.0, 28.9, 29.0, 65.7, 74.9, 112.7, 114.7, 123.7, 127.1, 129.4, 135.8, 137.9, 154.1; LRMS *m/z *(rel. int.): 244 (M^+^, 9.8), 230 (15), 229 (100); CH analysis calcd. for [C_16_H_21_O_2_] C 78.65, H 8.25, found C 78.19, H 8.04.

*1,4,4-Trimethyl-1,4,5,6-tetrahydro-2H-benzo[f]isochromen-9-ol* (**3c**). Yield: 71%, White solid; mp: 160–162 °C; IR (film): 3235, 2962, 2943, 1615, 1572, 1497, 834, 809 cm^−1^; ^1^H-NMR (200 MHz, CDCl_3_) δ: 1.21 (d, *J *= 6.8 Hz, 3H), 1.35 (s, 3H), 1.45 (s, 3H), 2.17–2.08 (m, 2H), 2.69–2.55 (m, 3H), 3.65 (dd, *J *= 11.1, 2.4 Hz, 1H), 3.93 (dd, *J *= 11.1, 3.3 Hz, 1H), 6.64 (dd, *J *= 7.8, 2.4 Hz, 1H), 6.79 (d, *J *= 2.4 Hz, 1H), 6.98 (d, *J *= 7.8 Hz, 1H); ^13^C-NMR (50 MHz, CDCl_3_) δ: 18.0, 24.2, 24.9, 27.69, 27.74, 28.9, 65.6, 75.4, 109,9, 112.8, 127.5, 128.1, 129.6, 135.1, 138.8, 154.6; LRMS *m/z *(rel. int.): 244 (M^+^, 9.3), 230 (15), 229 (100); HRMS [ESI(+)] calcd. for [C_16_H_20_O_2_+ Na]^+^ 267.1355, found. 267.1359.

*8-Methoxy-1,4,4-trimethyl-1,4,5,6-tetrahydro-2H-benzo[f]isochromen-9-ol* (**3d**). Yield: 42%. Pale yellow solid; mp: 141–143 °C; IR: 3396, 2967, 2924, 1509, 869, 809 cm^−1^; ^1^H-NMR (200 MHz, CDCl_3_) δ: 1.23 (d, *J *=7.2 Hz, 3H), 1.30 (s, 3H), 1.40 (s, 3H), 2.05–2.14 (m, 2H), 2.56–2.66 (m, 3H), 3.66 (dd, *J *= 2.2, 11.1 Hz, 1H), 3.89 (s, 3H), 3.89–3.96 (m, 1H), 5.60 (br, 1H), 6.69 (d, *J *= 12.2 Hz, 1H), 6.83 (d, *J *= 18.2 Hz, 1H); ^13^C-NMR (50 MHz, CDCl_3_) δ: 18.1, 24.1, 24.7, 28.0, 28.1, 29.2, 56.3, 65.7, 74.9, 106.1, 109.5, 110.5, 114.1, 126.4, 129.3, 129.4, 136.3, 144.0, 144.9; LRMS *m/z *(rel. int.): 274 (15.8), 260 (13.1), 259 (100); HRMS [ESI(+)] calcd. for [C_17_H_22_O_3_+ H]^+ ^275.1642, found 275.1648.

*1,4,4-Trimethyl-1,4,5,6-tetrahydro-2H-benzo[f]isochromene-8,9-diol* (**3e**). The reaction was performed following the general procedure of Section 4.3, but using NaH (23 mmol), EtSH (18 mmol), **2d** (0.144, 0.500 mmol). Yield: 76%. Pale yellow solid; mp: 122–123 °C; IR (film): 3605, 3533, 2876, 2828, 1619, 1601, 959, 939, 896, 687 cm^−1^; ^1^H-NMR (300 MHz, CDCl_3_) δ: 1.18 (d, *J *= 6.8 Hz, 3H), 1.32 (s, 3H), 1.42 (s, 1H), 2.08–2.13 (m, 2H), 2.55–2.62 (m, 3H), 3.64 (dd, *J *= 2.4, 11.2 Hz, 1H), 3.92 (dd, *J *= 3.2, 11.2 Hz, 1H), 3.67 (br, 2H), 6.67 (s, 1H), 6.81 (s, 1H); ^13^C-NMR (75 MHz, CDCl_3_) δ: 18.1, 24.1, 24.7, 28.0, 28.1, 29.1, 65.7, 110.3, 114.7, 127.2, 128.8, 129.3, 136.2, 141.9, 142.0; LRMS *m/z *(rel. int.):260 (M^+^, 10), 246 (13), 245 (100); HRMS [ESI(+)] calcd. for [C_16_H_19_O_3_+ Na]^+^ 283.1305, found 283.1316.

### 3.4. Antiproliferative Assays

Human tumor cell lines [U251 (glioma), MCF-7 (breast), NCI-ADR/RES (ovarian expressing phenotype multiple drugs resistance), 786–0 (renal), NCI-H460 (lung, non-small cells), PC-3 (prostate), OVCAR-03 (ovarian) and HT-29 (colon)] were kindly provided by Frederick Cancer Research & Development Center–National Cancer Institute–Frederick, MA, USA. HaCat cell line (immortalized human keratinocytes) was kindly donated by Dr. Ricardo Della Coletta, FOP–Unicamp. Stock cultures were grown in 5 mL of RPMI 1640 (GIBCO BRL, Life Technologies) supplemented with 5% of fetal bovine serum. Penicillin:streptomycin (1,000 μg mL^−1^:1,000 UI mL^‑1^, 1 mL L^−1^) was added to the experimental cultures.

Cells in 96-well plates (100 μL cells/well) were exposed to various concentrations of compounds **2a–d** and **3a–d** diluted in DMSO (0.25, 2.5, 25 and 250 μg/mL) at 37 °C, 5% of CO_2_ for 48 h. The final concentration of DMSO did not affect the cell viability. Afterwards cells were fixed with 50% trichloroacetic acid and cell proliferation determined by spectrophotometric quantification (540 nm) of cellular protein content using sulforhodamine B assay[20]. Doxorubicin (0.025–25 mg/mL) was used as positive control. Three measurements were obtained at the beginning of incubation (time zero, T_0_) and 48 h post-incubation for compound-free (C) and tested (T) cells. Cell proliferation was determined according to the equation 100 × [(T − T_0_)/C − T_0_], for T_0_ < T ≤ C, and 100 × [(T − T_0_)/T_0_], for T ≤ T_0_ and a concentration-response curve for each cell line was plotted and, from these curves, GI50 (concentration causing 50% growth inhibition) and TGI (concentration that promotes total growth inhibition) were determined by means of non-linear regression analysis using software ORIGIN 8.0 (OriginLab Corporation) [20,21] The average activity (mean of log GI_50_) of each compound tested was also determined using MS Excel software. Compounds were regarded as inactive (mean > 1.5), weakly (1.1 < mean < 1.5), moderately (0 < mean < 1.1) or potently (mean < 0) active on basis of the NCI criteria for the mean of logGI_50_[19].

## 4. Conclusions

In conclusion, the efficient synthesis of nine new populene D analogues **2a–d** and **3a–e** was performed using an iodine catalyzed Prins cyclization as key transformation. The antiproliferative activity of these compounds against several cancer cell lines was investigated. This evaluation demonstrated that isochromene **3e** is the most active compound on the cell lines used. Although the populene D analogues do not display potent antiproliferative activity, we think this work might inspire the discovery of new highly active isochromenes.

## Supplementary Materials

Supplementary materials can be accessed at: http://www.mdpi.com/1420-3049/17/8/9621/s1.
